# Supplementary material for: The Genetic Control of SEEDSTICK and LEUNIG-HOMOLOG in Seed and Fruit Development: New Insights into Cell Wall Control
Source: Plants (Basel). 2022 Nov 17;11(22):3146. doi: 10.3390/plants11223146 (PMC9698089; doi:10.3390/plants11223146)
Supplement: Supplementary file 1 [file plants-11-03146-s001.zip › plants-1958454-supplementary figures.pdf]

Table S1: **T-DNA insertion lines and corresponding WT used in the work**

**Stocks**	**Mutated gene**	**Locus**	**Germplasm**	**Lab origin**	**Publication origin**	**Link source**
Ws-2				Tamara Western lab, Montreal (CA)		
bxl1-1	*BXL1*	AT5G49360	CS16299	Tamara Western lab, Montreal (CA)	Arsovski *et al.*, 2009	https://www.ncbi.nlm.nih.gov/pmc/articles/PMC2705025/
Col-0				Own lab, Milan (IT)		
cesa5-2	*CESA5*	AT5G09870	SALK_023353	Chris Somerville lab, Stanford (USA)	Persson *et al.*, 2007	https://pubmed.ncbi.nlm.nih.gov/17878302/
fei2-2	*FEI2*	AT2G35620	CS2106721-SALK_044226	Joe Kieber lab, Chapel Hill (USA)	Harpaz-Saad *et al.*, 2012	https://www.ncbi.nlm.nih.gov/pmc/articles/PMC3405700/
fly1.1	*FLY1*	AT4G28370	CS67936	George Haughn lab, Vancouver (CA)	Voiniciuc *et al.*, 2013	https://pubmed.ncbi.nlm.nih.gov/23482858/
fly1.3	*FLY1*	AT4G28370	SALK_144822	George Haughn lab, Vancouver (CA)	Voiniciuc *et al.*, 2013	https://pubmed.ncbi.nlm.nih.gov/23482858/
gl2-8	*GL2*	AT1G79840	SALK_130213C	NASC collection, Nottingham (UK)	Western *et al.*, 2004	https://pubmed.ncbi.nlm.nih.gov/14701918/
luh-3	*LUH/MUM1*	AT2G32700	SALK_107245C	NASC collection, Nottingham (UK)	Saez-Aguayo *et al.*, 2013	https://academic.oup.com/plcell/article/25/1/308/6097743
per36-1	*PER36*	AT3G50990	SAIL_194_G03	Ikuko Hara-Nishimura lab, Kyoto (JP)	Kunieda *et al.*, 2013	https://academic.oup.com/plcell/article/25/4/1355/6100529
pmei6-1	*PMEI6*	AT2G47670	SM3.19557	Helen North lab, Versailles (FR)	Saez-Aguayo *et al.*, 2013	https://academic.oup.com/plcell/article/25/1/308/6097743
stk-2	*STK*	AT4G09960		Own lab, Milan (IT)	Pinyopich *et al.*, 2003	https://pubmed.ncbi.nlm.nih.gov/12840762/

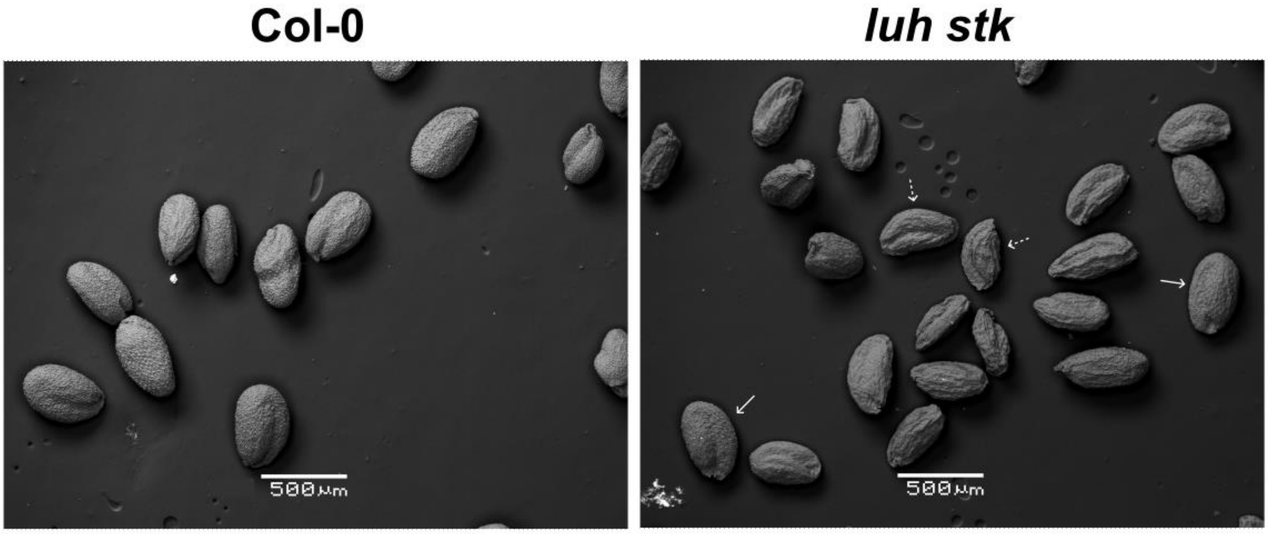

Figure S1: **Scanning Electron Microscopy (SEM) pictures of seeds from Columbia (WT) and *luh stk* double mutant.** In *luh stk* image normal arrows indicate WT-like seeds, dotted arrow indicate seeds with “wrinkled” phenotypes. Scale bar = 500 µm.

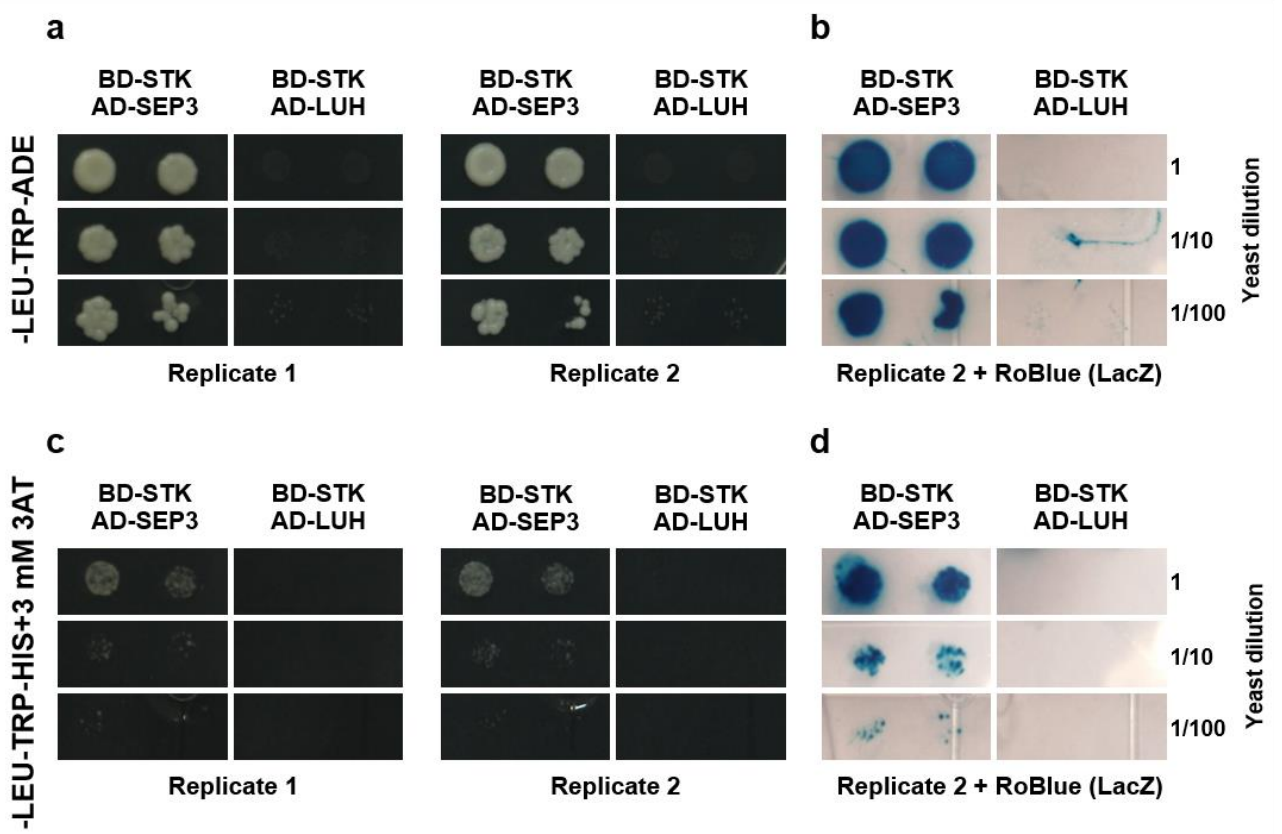

Figure S2: **Yeast Two-Hybrid assay to test the LUH-STK protein interaction.** The direct interaction between STK and LUH was tested using a yeast two-hybrid assay. The interaction STK-SEPALLATA 3 (SEP3) was used as a positive control. Two different colonies were used for the experiments with one technical replicate. Three yeast dilutions were used for the inoculation on the interaction media. The picture shows yeast growth seven days after inoculation and incubation at 22°C.

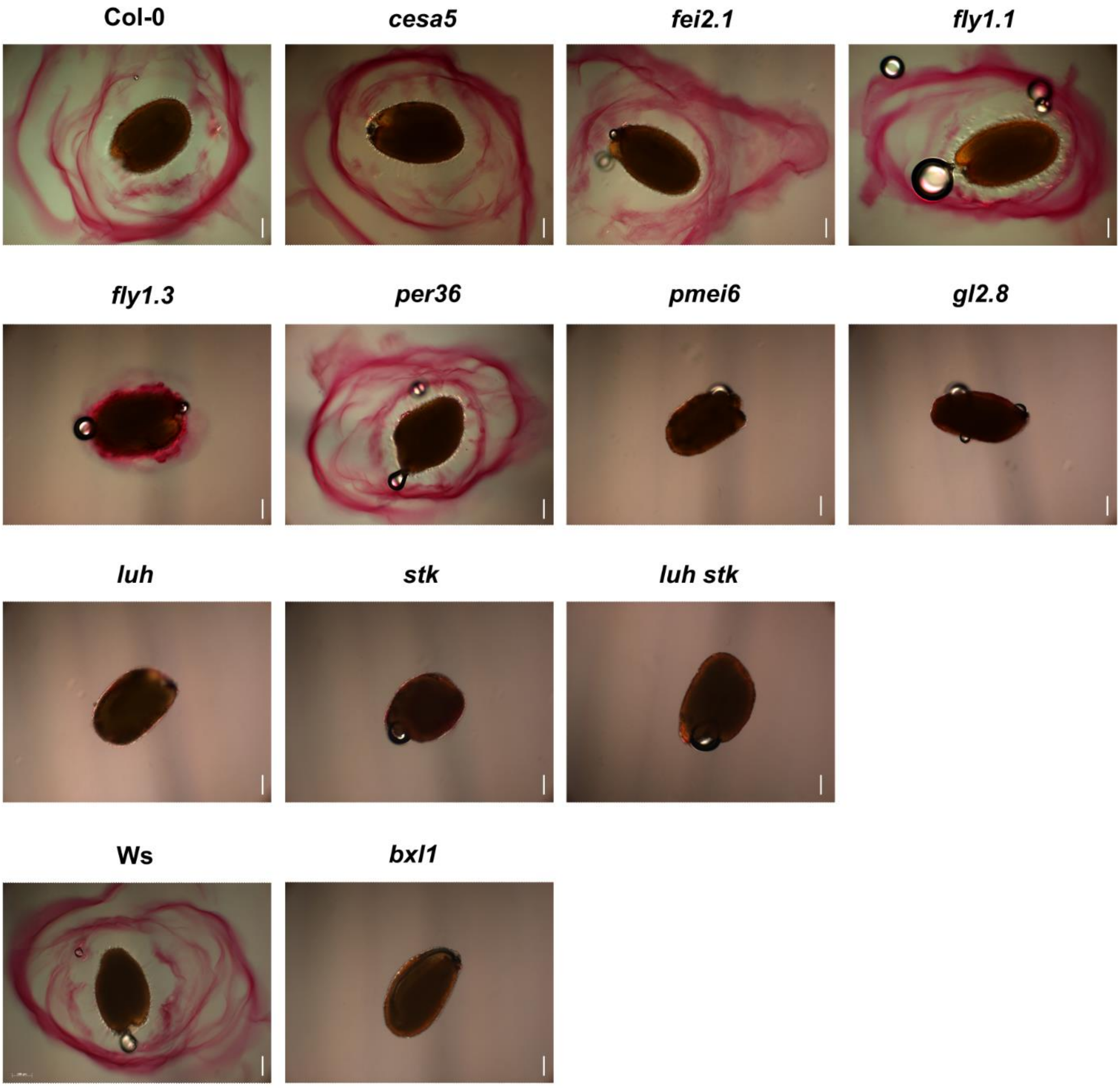

Figure S3: **Ruthenium red staining in seeds from the cell wall mutants for the mucilage extrusion analysis.** Stereomicroscope images of *cesa5*, *fei2.1*, *fly1.1*, *fly1.3*, *per36*, *pmei6*, *gl2.8*, *luh*, *stk*, *luh stk* and *bxl1* compared to the corresponding WT (Col-0 and Ws). Scale bar = 100 µm;

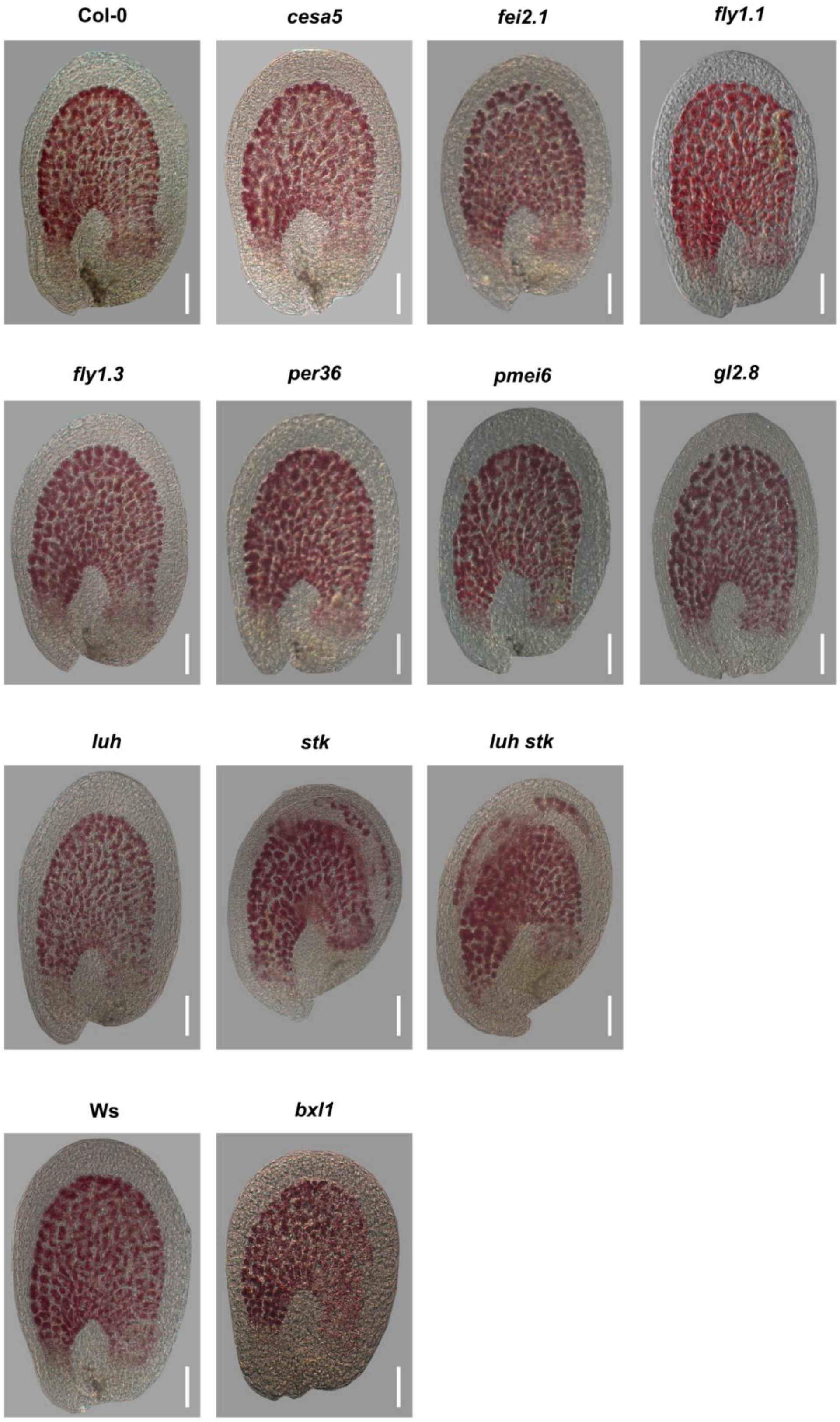

Figure S4: **Vanillin staining in seeds from the cell wall mutants for the proanthocyanidin accumulation analysis.** Microscope images of *cesa5*, *fei2.1*, *fly1.1*, *fly1.3*, *per36*, *pmei6*, *gl2.8*, *luh*, *stk*, *luh stk* and *bxl1* compared to the corresponding WT (Col-0 and Ws). Scale bar = 50 μm.
